# A gene network regulated by FGF signalling during ear development

**DOI:** 10.1038/s41598-017-05472-0

**Published:** 2017-07-21

**Authors:** Maryam Anwar, Monica Tambalo, Ramya Ranganathan, Timothy Grocott, Andrea Streit

**Affiliations:** 1 Department of Craniofacial Development & Stem Cell Biology, King’s College London, London, SE1 9RT UK; 2 Imperial College London, Institute of Clinical Sciences, Faculty of Medicine, South Kensington Campus, London, SW7 2AZ UK; 3 Present Address: The Francis Crick Institute, 1 Midland Road, Kings Cross, London, NW1 1AT UK; 4 School of Biological Sciences, University of East Anglia, Norwich, NR4 7TJ UK

## Abstract

During development cell commitment is regulated by inductive signals that are tightly controlled in time and space. In response, cells activate specific programmes, but the transcriptional circuits that maintain cell identity in a changing signalling environment are often poorly understood. Specification of inner ear progenitors is initiated by FGF signalling. Here, we establish the genetic hierarchy downstream of FGF by systematic analysis of many ear factors combined with a network inference approach. We show that FGF rapidly activates a small circuit of transcription factors forming positive feedback loops to stabilise otic progenitor identity. Our predictive network suggests that subsequently, transcriptional repressors ensure the transition of progenitors to mature otic cells, while simultaneously repressing alternative fates. Thus, we reveal the regulatory logic that initiates ear formation and highlight the hierarchical organisation of the otic gene network.

## Introduction

Unravelling the structure of regulatory circuits that control development provides mechanistic insight into the assembly of a body plan and functional organs. Experimental perturbation combined with network inference offers a powerful approach to establish the topology of regulatory networks and to predict the mechanisms underlying biological processes. Here we use the vertebrate inner ear as a model to study how signalling events initiate a developmental programme and how this programme is subsequently stabilized. The inner ear arises from a simple epithelium, the otic placode, which in amniotes is first visible at the 10 somite stage (ss) as a sheet of columnar cells next to rhombomeres 5 and 6 of the hindbrain^1, 2^. The placode then invaginates forming the otic cup, which separates from the surface ectoderm to generate the otic vesicle. The vesicle gradually acquires the architecture of the adult inner ear through morphogenetic changes accompanied by the differentiation of a large number of specialised cell types.

At placode stages, cells are committed to inner ear fate, but prior to this, are part of a progenitor pool with the potential to contribute to other sense organs and to cranial sensory ganglia. These precursors are confined to a band of ectoderm that surrounds the anterior neural plate, which has been termed the pre-placodal region (PPR)^3–7^. Under the influence of fibroblast growth factor (FGF) signalling progenitor potential is restricted and cells become specified as otic-epibranchial precursors (OEPs)^8–19^. Initially FGFs emanate from the underlying mesoderm like FGF19 in chick and FGF10 in mouse, while later hindbrain derived FGF3 contributes to OEP induction^9, 18^. It has been suggested that thereafter reduction of FGF signalling maybe required^20^ before a combination of Wnt and Notch signalling promotes otic identity^20–22^. Thus, complex signalling events gradually commit sensory progenitors to the otic lineage.

Downstream of these signals a number of transcription factors are activated, which in turn are required for otic specification^1^. However, only a few factors respond to FGF signalling^8, 14, 17, 23^, and among these many become expressed only at later stages (e.g. *Foxg1*, *Sox10*), after otic cells are specified and maintain their character in the absence of additional signalling^24, 25^. These findings suggest that a small regulatory network may act immediately downstream of FGF to stabilise OEP identity prior to otic commitment. To explore this, we took advantage of an established *in vitro* system to modulate FGF signalling over time, quantified changes of more than 100 genes expressed in otic and other placodal cells and then used a Random Forests technique to infer a gene regulatory network that models FGF action. This systems approach identifies a circuit of positive feedback loops that stabilises OEP identity in response to FGF, followed by reciprocal inhibitory interactions to refine time and space of otic gene expression and to repress alternative fates.

## Results

### Sensory progenitors transiently activate OEP genes in the absence of FGF signalling

At head fold stages, all placode progenitors are specified as lens, irrespective of their later fate: when cultured in isolation they initiate the lens programme and the induction of other placodes requires its repression by local inducing signals^26, 27^. The first step in otic induction is the formation of otic-epibranchial progenitors (OEPs) in the posterior pre-placodal region (pPPR) mediated by FGF signalling^1, 28, 29^. Here we explore the temporal dynamics in response to FGF during OEP induction using a well-characterised *in vitro* assay^25^.

**Figure 1:**
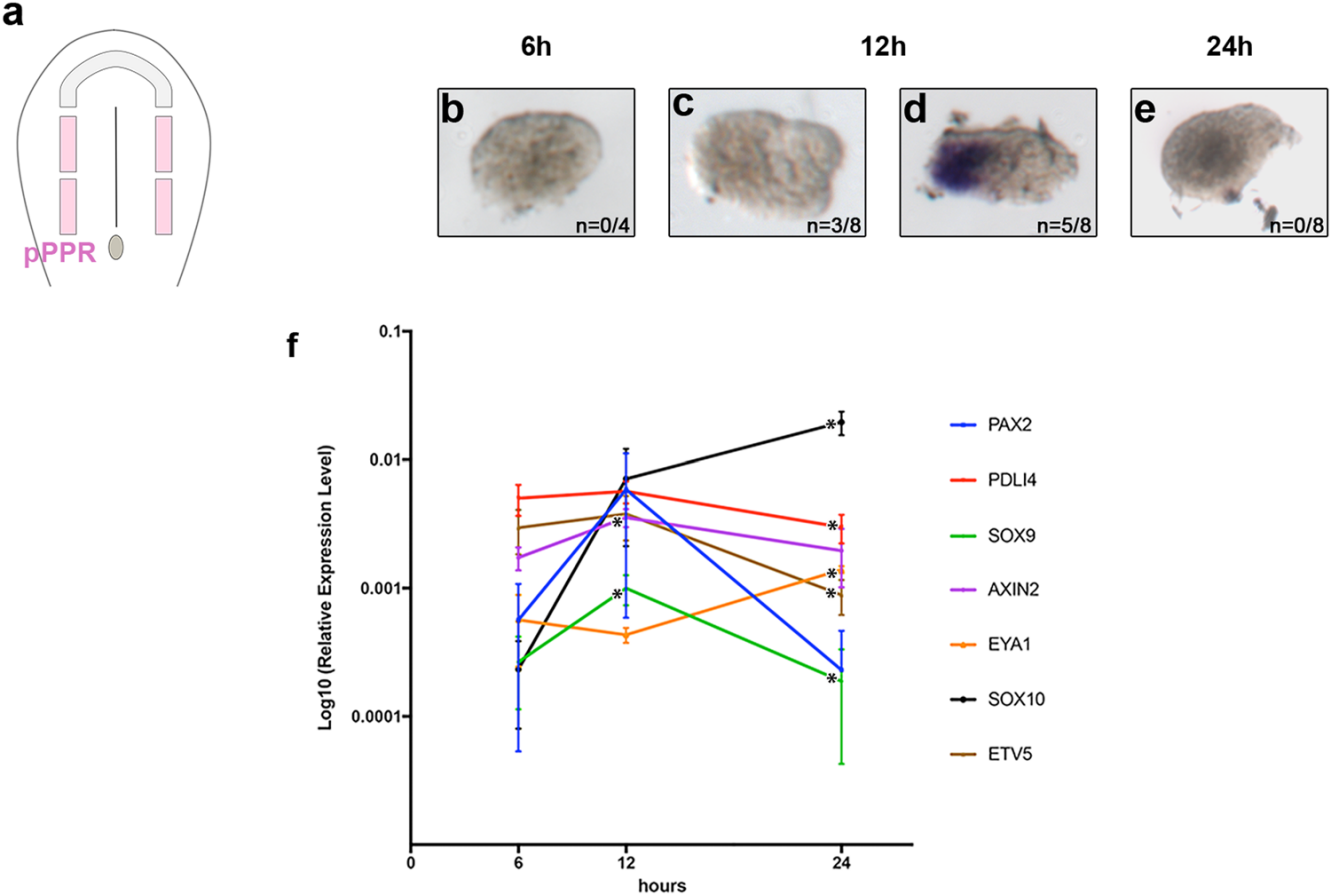
Transient expression of otic markers. Cultured pPPR ectoderm (pink) (**a**) was assessed for *Pax2* expression (**b**–**e**); 62% of the explants are *Pax2* ^+^ after 12 hours’ culture (**c**,**d**). NanoString analysis shows a few other otic genes with a similar profile, while *Sox10* and *Eya1* increase at 24 hrs (**f**). Error bars in f represent the standard error; asterisk: statistically significant change.

To establish a baseline we first characterised dynamic changes of gene expression in posterior PPR explants in the absence of FGF. Posterior PPR from head fold stages (HH6) was cultured in isolation for 6, 12 or 24 hours (Fig. 1a–e). Gene expression in 7–10 pooled explants was analysed by NanoString using a probe set containing recently identified^31^ and known PPR transcripts, otic markers and transcripts specific for other placodes as well as for the neural plate and neural crest, direct targets of various signalling pathways and housekeeping genes (see supplementary file 1).

The transcription factor *Pax2*, one of the earliest OEP-specific genes, is considered to be FGF dependent^9–11, 15, 18, 20, 32–40^. Surprisingly we find that its expression is activated at 12 hours, but subsequently lost (Fig. 1b–e; Supplementary Fig. S1, cluster 3). We verified this observation using *in situ* hybridisation: after 6 hours *in vitro*, *Pax2* expression is not observed in isolated posterior PPR explants (n = 0/4 explants; Fig. 1b), but is clearly detectable at 12 hrs (5/8 explants; 62%; Fig. 1c,d), but not at 24 hrs (0/8 explants; Fig. 1e). Thus, *Pax2* is transiently upregulated even in the absence of otic inducing signals. This observation prompted us to investigate whether other otic genes show a similar behaviour. We find that some otic transcripts like *Etv5*, *PDL14*, *Axin2* and *Sox9* behave similar to *Pax2* with upwards trend at 12 hrs decreasing again at 24 hrs, while others like *Eya1* and *Sox10* are enhanced at 24 hours’ culture (Fig. 1f; note: not all changes are statistically significant). However, during the entire culture period lens and anterior transcripts (*Pax6*, *Otx2*, *Dlx5*, *Dlx6*; supplementary data 2) remain expressed at relatively high levels consistent with these explants being lens specified^26^. Although no FGFs are known to be expressed in the pPPR (see e.g. ref. 30), it is possible that placode progenitors have some residual FGF activity indicated by low levels of *Etv5* expression at the time of explanting^41^, which however is not sufficient to complete otic induction. Together, these results suggest that posterior PPR cells may have an autonomous tendency to form OEPs, but do not realise this potential in the absence of additional signals.

### A genetic hierarchy downstream of FGF signalling

**Figure 2:**
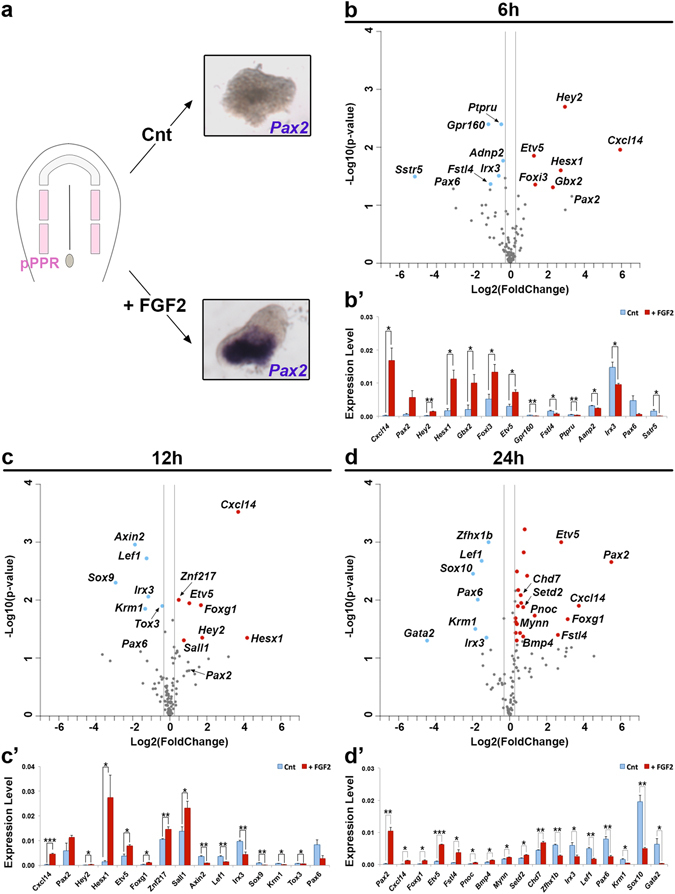
FGF2-regulated transcripts. When cultured in isolation pPPR explants do not express *Pax2* after 24 hrs, while addition of FGF2 induces *Pax2* (**a**). Changes in gene expression after 6, 12 and 24 hrs FGF-treatment was assessed by NanoString; results are plotted using Log2 transformed fold change (+FGF2/Control) (x-axis) and −Log10 (p-value) (y-axis) (**b**–**d**). A fold change of 1.5 or 0.25 (grey lines) and a p-value < 0.05 were used as threshold; transcripts not passing these thresholds are shown in grey and significantly up- and downregulated genes are shown in red and blue, respectively. (**b’**–**d’**) Bar charts showing transcripts with significant changes; controls in blue and FGF2-treated in red. Error bars represent the standard error. Asterisks (***, ** and *) indicate significant differences (0.001, 0.01 and 0.05, respectively).

In chick PPR explants, FGF2 mimics the activity of the endogenous OEP promoting signal, FGF19^9, 11, 14, 33, 42^. To explore the dynamics in response to FGF signalling PPR explants (0 ss) freed from all surrounding tissues were cultured in the presence or absence of FGF2 for 6, 12 or 24 hours (Fig. 2a). Changes in the expression of 126 transcripts were quantified using NanoString (Supplementary File 2). To identify groups of genes with a similar FGF2-response profiles we performed hierarchical clustering of the log transformed gene expression levels (Supplementary Fig. S1) revealing 11 main clusters (denominated C1-C11). Transcripts in clusters C1, C5, C6 and C11 do not change in control or in FGF-treated conditions, while genes in other clusters are activated (C3, C7) or repressed (C10). These results indicate that FGF alone can only induce a small number of assayed transcripts, as also described previously^23^. Indeed, 6 hours of FGF exposure leads to the induction of only 6 transcripts (Fig. 2b) including the FGF target *Etv5* ^41^, the pPPR genes *Foxi3* (Supplementary Fig. S2)^43^, *Gbx2* (Supplementary Fig. S2)^44^ and *Hey2* (Supplementary Fig. S2), the late otic gene *Hesx1* ^45^ (Supplementary Fig. S5) and the chemokine *Cxcl14*, which is normally expressed along the medial edge of the OEP domain (Supplementary Fig. S2). Except for *Hey2* (cluster C2) these genes cluster together in C3. 12 hrs after FGF2 exposure genes normally expressed at otic placode stages are initiated (*Znf217* [C6], *Sall1* [C1]; Fig. 2c; Supplementary Fig. S1). Finally, after 24 hrs, *Pax2* is significantly enhanced compared to controls together with a few signalling components (*pNoc*, *BMP4*, *Fstl4;* Supplementary Fig. S2) and chromatin remodelers (*Chd7*, *Setd2*; Fig. 2d; Supplementary Fig. S2).

While activating OEP associated genes, FGF exposure also leads to repression of genes normally absent from the otic territory. The majority of these transcripts fall into two clusters, C2 (*Sstr5*, *Kremen1*, *Tox3*, *Ptpru* and *Gpr160*) and C10 (*Pax6*, *Dlx5*/*6* and *Lef1*) (Supplementary Fig. S1) and largely characterise the anterior PPR (future lens/olfactory; Supplementary Fig. S2)^26, 30^. This confirms the previous observation that FGF signalling is important to repress lens specification in non-lens ectoderm^26^. Finally, FGF also modulates other signalling pathways. While the WNT targets *Lef1* (12 and 24 hrs) and *Axin2* (12 hrs) are downregulated, the Notch target *Hey2* ^46^ increases. Likewise, FGF appears to modulate BMP signalling: the BMP antagonist *Fstl4* is first repressed (6 hrs) and then induced (24 hrs) together with *Bmp4* (Fig. 2b–d).

In summary, FGF signalling induces only a subset of the otic transcripts investigated here^23^ and does so in a temporal hierarchy. First, FGF enhances transcripts already expressed in the posterior PPR, followed by the initiation of OEP and late otic genes more downstream. At the same time, FGF activity ensures the repression of alternative fates.

### FGF regulates *Etv5* and *Pax6* directly

**Figure 3:**
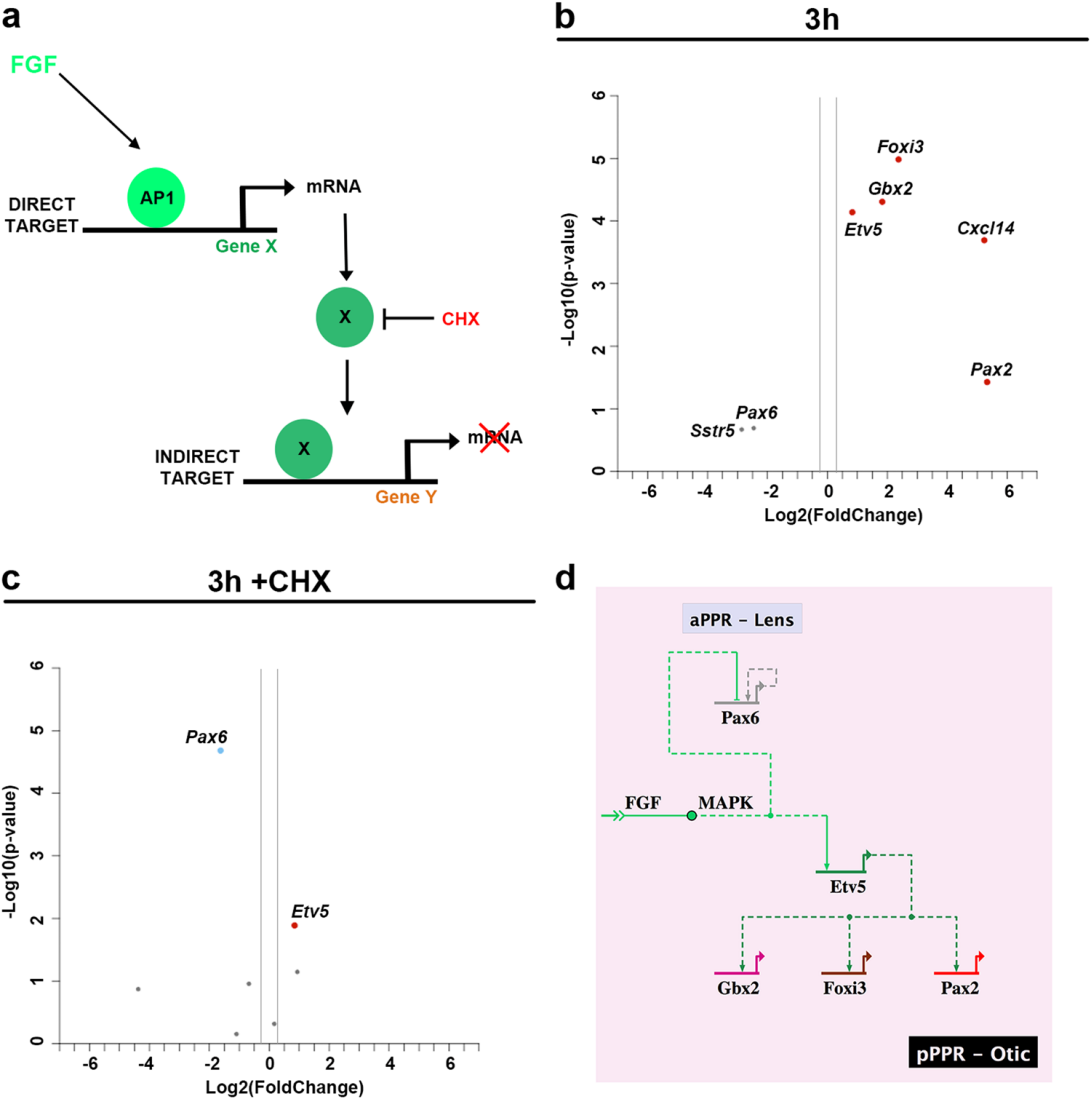
Direct FGF targets. FGF signaling directly regulates gene expression (Gene X) via AP1 (Jun/Fos complex), thereafter Gene X may activate indirect targets (Gene Y). Addition of the protein synthesis blocker cycloheximide (CHX) allows the identification direct FGF targets (**a**). After 3 hrs culture, gene expression in control and FGF treated pPPR explants was quantified by RT-qPCR (**b**). Explants were treated with CHX, in the presence or absence of FGF, and gene expression was analyzed RT-qPCR (**c**). Genes significantly up- and downregulated (≥1.5 or ≤0.25-fold change) are indicated in red and blue, respectively (p-value < 0.05). Etv5 and Pax6 are the only direct targets. (**d**) Simple network showing gene activation/repression downstream of FGF.

Are all 6 hr-induced genes direct FGF targets? After 3 hrs of FGF2 treatment early OEP genes (*Etv5*, *Cxcl14*, *Gbx2*, *Foxi3* and *Pax2*) are upregulated and lens genes (*Pax6* and *Sstr5*) are repressed (Fig. 3b). To identify the direct targets, the same experiment was carried out in the presence of the translation inhibitor cycloheximide (CHX; Fig. 3a)^47^. Among the upregulated transcripts tested, only *Etv5* appears as a direct FGF target (Fig. 3c), while *Pax6* is the only factor repressed in the absence of protein synthesis (Fig. 3c). Therefore, the most parsimonious explanation for how FGF signalling activates the downstream network is that Etv5 regulates other upregulated factors, while Pax6 initiates the repressive cascade (Fig. 3d).

### FGF activity is required for few mesoderm-induced OEP genes

**Figure 4:**
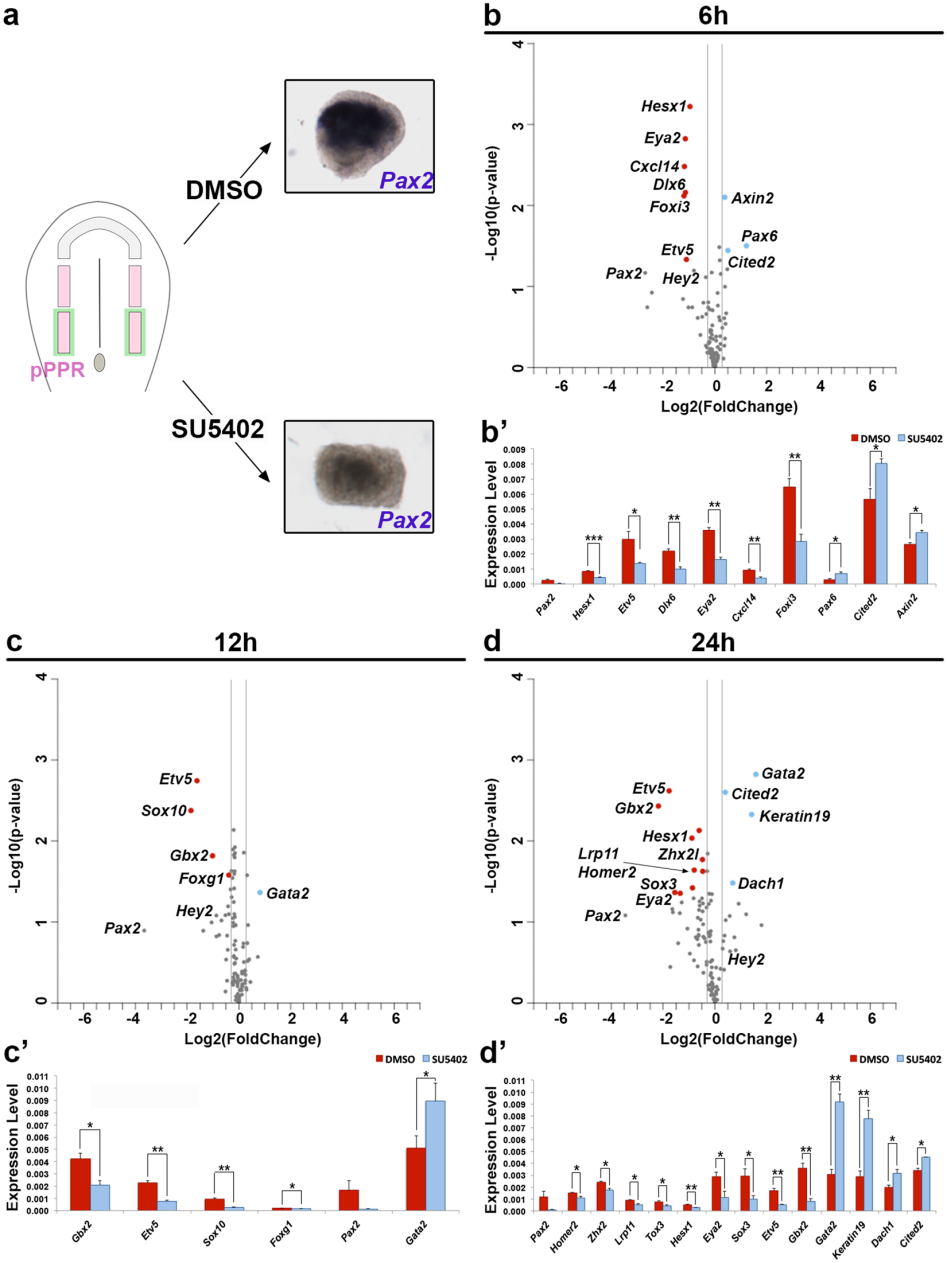
Requirement of FGF signalling for mesoderm induced otic genes. 0 ss pPPR ectoderm (pink) was dissected together with the underlying mesoderm (green), the endogenous source of FGF. Inhibition of FGF signalling by SU5402 inhibits *Pax2* expression (**a**). Changes in gene expression was assessed after 6, 12 and 24 hrs by NanoString. Log2 transformed fold change (SU5402/DMSO) are plotted against –Log10 (p-value) (**b**–**d**). A 1.5 and 0.25-fold change was used as threshold (grey lines); transcripts not passing these thresholds are shown as grey dots. Significantly up- and down-regulated genes are shown in red and blue, respectively (p- value < 0.05). (**b’**–**d’**) Bar chart showing transcripts with significant changes; controls in red and SU5402-treated in blue. Error bars represent the standard error. Asterisks (***, ** and *) indicate significant differences (0.001, 0.01 and 0.05, respectively).

*In vivo*, the head mesoderm is one of the FGF sources required for OEP induction^33, 48^. To investigate how the requirement for mesoderm-derived FGF changes over time we compared posterior PPR co-cultured with the head mesoderm in the presence of DMSO (control) or the FGFR inhibitor SU5402 (experimental; Fig. 4a) and assessed changes in gene expression after 6, 12 and 24 hrs using NanoString.

After 6 hours’ culture, 4 out of 6 mesoderm-induced transcripts depend on FGF signalling: the expression of *Etv5*, *Foxi3*, *Hesx1*, *Cxcl14* is lost in the presence of the inhibitor (Fig. 4b). After 12 hrs another 6hr-induced gene, *Gbx2*, also emerges as FGF-dependent (Fig. 4c), while *Hey2* induction is FGF independent. In addition, expression of the PPR genes *Eya2* (6 hrs & 24 hrs; Fig. 4b,d) and *Dlx6* (6 hrs; Fig. 4b) decreases in the presence of SU5402, suggesting that their maintenance requires FGF signalling. In contrast, repression of the anterior PPR genes tested is largely FGF independent and only *Pax6* inhibition initially requires this pathway. Other anterior transcripts like *Otx2*, *pNoc*, *Six3* and *Sstr5* are decreased by mesodermal signals even when FGF signalling is reduced, as are *Dlx5* and *-6* at 24 hrs (Supplementary File 2).

After 12 and 24 hours’ culture other additional FGF-dependent factors emerge among them the otic placode genes *Sox10* and *Foxg1* (12 hrs; Fig. 4c) as well as *Sox3*, *Zhx2 l* and *Homer2*. Among the transcripts repressed by both the mesoderm and FGF, the non-neural ectoderm marker *Gata2* together with epidermal *Keratin19* stand out as the few genes whose repression after 12 hrs and 24 hrs, respectively, requires FGFR activity (Fig. 4c,d).

Together these results indicate that only those transcripts rapidly induced by FGF2 or the head mesoderm depend on FGF activity, while only a few late onset genes do. Likewise, repression of anterior character appears to be largely FGF independent, while inhibition of epidermal character may be mediated by FGF. Overall, these observations suggest that other signals must cooperate with mesoderm derived FGF to promote OEP specification.

### Gene network inference and clustering identify sub-networks in the OEP programme

**Figure 5:**
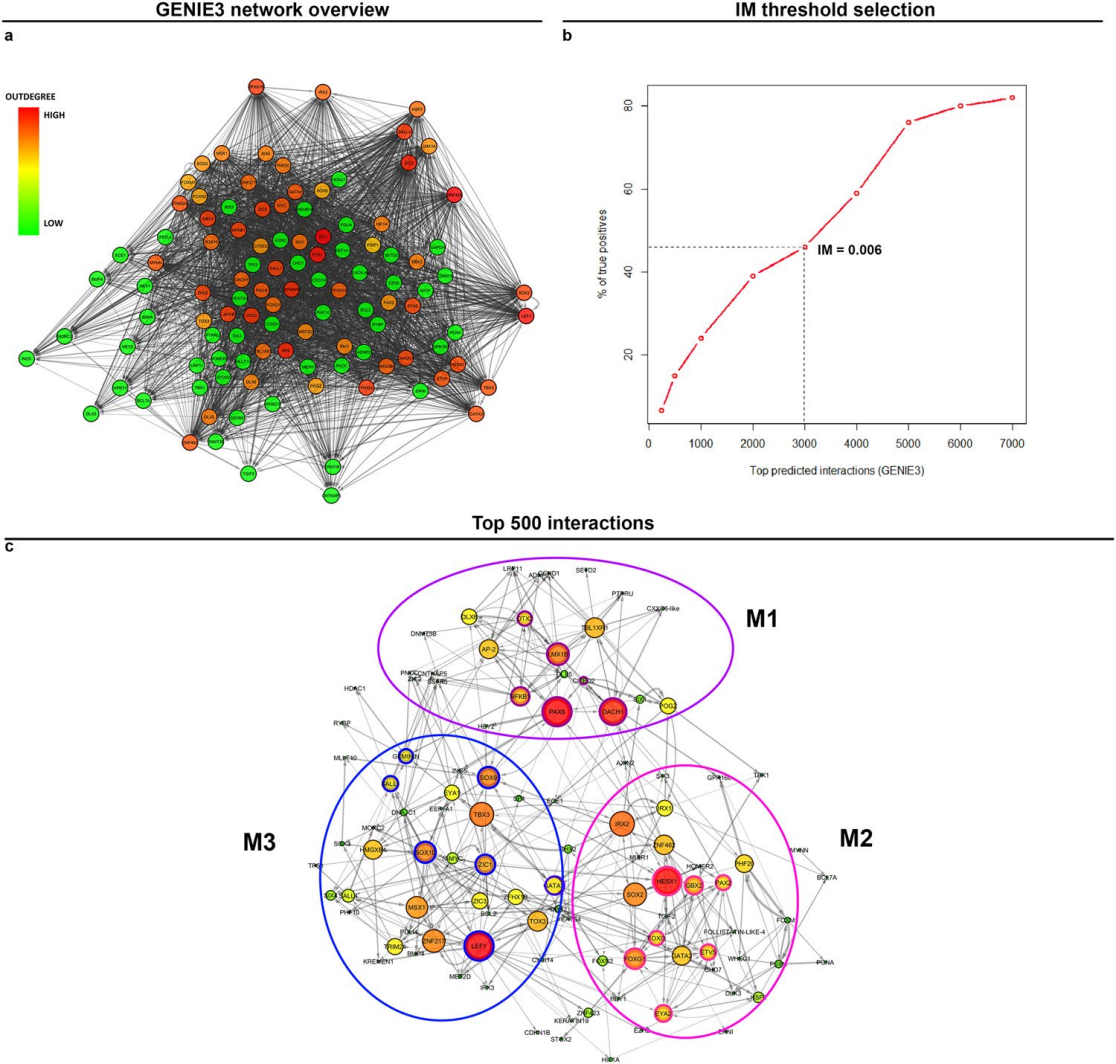
Network inference using GENIE3 reveals different modules. Using GENIE3, a directed network of interactions was predicted among the genes in NanoString data. Cytoscape view of the network where nodes are coloured according to their out-degrees (interactions emerging from each node); higher out-degrees are colour-coded in red and low out-degrees in green (**a**). To analyze accuracy of predictions, the percentage of true positives (known interactions from literature) retrieved by GENIE3 were plotted against the total number of predictions at various IM thresholds; a cut-off of IM >= 0.006 was selected (**b**). Analysis of top 500 predicted interactions above the threshold reveals three modules (**c**): M1 corresponds to FGF-repressed genes (anterior genes: nodes encircled in purple), M2 corresponds to genes initiated by FGF rapidly (nodes encircled in pink) and M3 to late FGF-response genes (nodes encircled in blue).

The above results reveal the molecular hierarchy downstream of FGF signalling as pPPR cells make the transition to OEPs. It is likely that direct FGF targets and early response genes (*Etv5*, *Gbx2*, *Foxi3*, *Hey2*) are at the top of this hierarchy providing input for late response genes. To explore this possibility in an unbiased way we used GENIE3, a Random Forest machine-learning algorithm, to infer a gene regulatory network (GRN) from the NanoString expression data. GENIE3 determines the importance of each factor within the network (regulator) in explaining the expression profile of a given target (by calculating importance measure: IM). To select an appropriate IM threshold the trade-off between recovery of true positives (based on data from the literature) and the number of overall predicted interactions was assessed (Fig. 5b); as the sensitivity of recovery drops, the threshold was set to 0.006. This results in a directed network of 3000 interactions and 109 nodes (Fig. 5a). To focus on the predictions with higher significance (larger IM values), the predicted interactions were ranked according to IM and the top 500 interactions were analysed in detail in Cytoscape (Fig. 5c; Supplementary File 3).

The predicted network topology reveals three modules termed M1, M2 and M3 (Fig. 5c) each containing genes with a similar response to FGF signalling: module M1 comprises genes repressed by FGF, many of which are normally expressed in the anterior PPR, module M2 contains early FGF response genes, while M3 includes factors regulated after 12 or 24 hours of FGF exposure. Node size in Fig. 5c indicates the centrality of each node within a module (see below).

**Figure 6:**
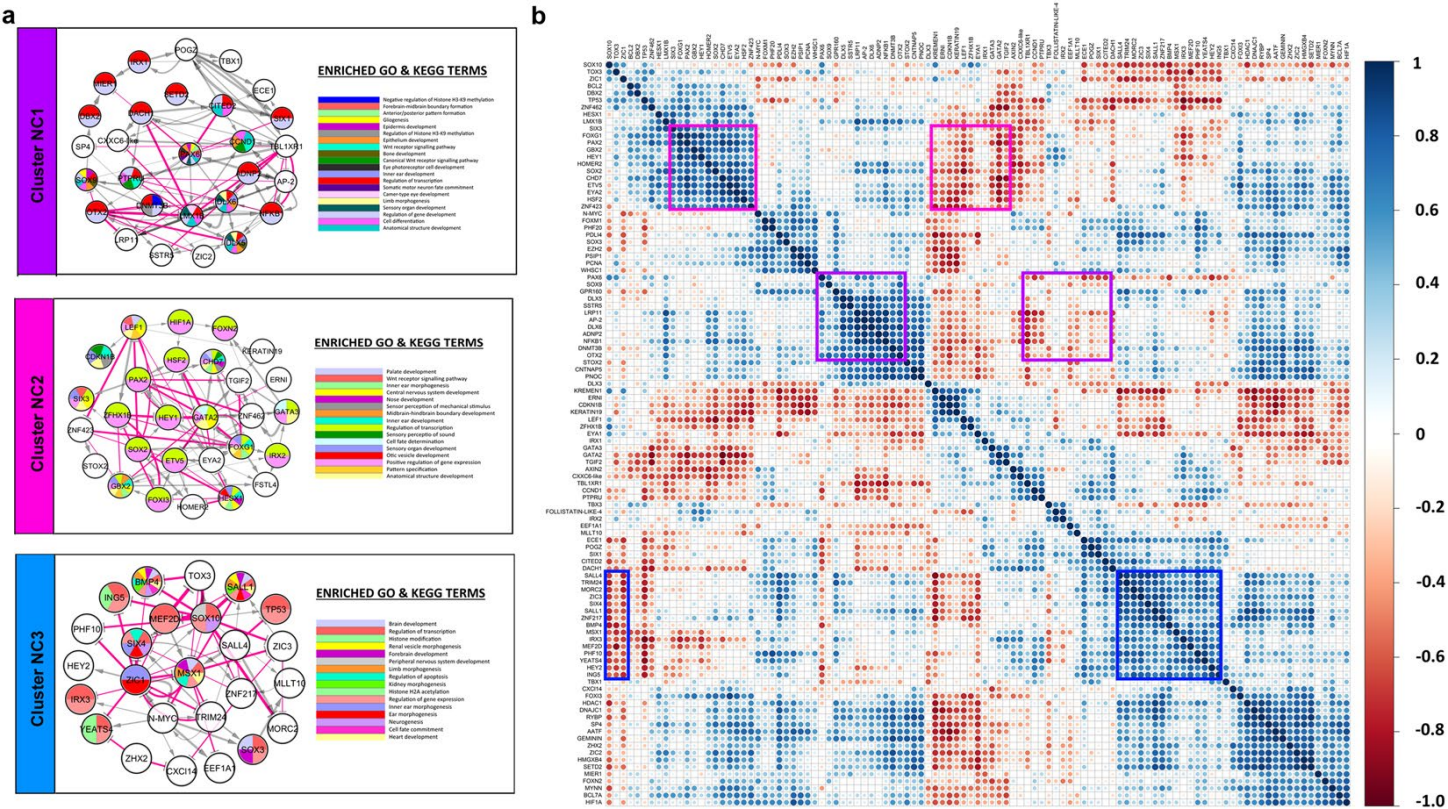
Community clustering of the top 500 GENIE3 predicted interactions identifies sub-networks in response to FGF. Clustering of the top 500 interactions in the predicted NanoString network using Newman’s community clustering (GLay Plugin in Cytoscape) confirms network modularity reveals 5 clusters (Clusters NC4 and NC5 are shown in Fig. S3). Each cluster was mapped to enriched GO and KEGG terms (P-value < 0.05) and nodes coloured accordingly (**a**). Genes that do not map to any terms are coloured white. Repressive (pink) and activating interactions were determined from the Pearson’s correlation coefficient values between the NanoString genes. Edges are weighted according to IM values. Cluster NC1 includes anterior genes that respond negatively to FGF with some corresponding GO terms including eye development and anterior/posterior pattern formation. Cluster NC2 corresponds to OEP and otic genes that respond positively to FGF with corresponding terms including inner ear development and sensory perception of sound. Cluster NC3 contains genes that respond to FGF later. Pearson’s correlation coefficient was calculated between all pairs of genes in the NanoString data and plotted as a heatmap (**b**). Clusters NC1-3 are highlighted as purple, pink and blue boxes in the heatmap. The colour key on the right indicates the correlation coefficient with dark blue corresponding to 1 and dark red to −1. Dot sizes in the heatmap correspond to the strength of correlation with 1 and −1 having the largest size.

To investigate the modularity of the emerging network, Newman’s community clustering was performed^49^ using the top 500 interactions of GENIE3 network (Supplementary File 3). This approach identifies gene hubs consisting of more connections within each hub than to the rest of the network. Five clusters (Newman’s cluster NC1-5) were identified consisting of 11 to 28 nodes and 19 to 92 edges (Fig. 6a, Supplementary Fig. S3); of these three clusters (NC1-3) correlate well with GENIE3 modules M1-M3 (see below). Both GENIE3 and Newmann’s community clustering predict groups of genes that may form a molecular sub-circuit and thus underlie a specific biological event (e.g. ‘early response to FGF’), however, they do not identify the most central genes in each circuit. We therefore calculated betweenness centrality for each node of the network as it provides a measure for the most connected nodes within a cluster (Fig. 5c). We displayed this in the GENIE3 network: large nodes show high betweenness centrality and are thus more central to the network. This analysis identifies four factors (Pax6, Dach1, Lef1 and Hesx1) as the most central nodes, as well as a larger number of slightly less well-connected nodes. These factors may play an important role during OEP specification in response to FGF signalling.

Finally, neither GENIE3 nor Newmann’s community clustering indicate whether the predicted interactions represent activation or repression of target genes. We therefore calculated Pearson’s correlation coefficient between all transcripts on the NanoString probe set (Fig. 6b: members of clusters NC1, NC2 and NC3 highlighted) assuming that positive correlations represent activating interactions, while negative correlations represent repressive interactions (Fig. 6a).

Next we examined the components of each cluster emerging from Newman’s community clustering and compared them to the GENIE3 modules. Cluster NC1 (Fig. 6a, NC1; Fig. 6b purple box) largely contains anterior PPR genes (*Pax6*, *Sstr5*, *Nfkb1*, *Dlx5*, *Dlx6*), similar to GENIE3 module M1. GO and KEGG term analysis (P-value < 0.05) reveals an enrichment of terms like anterior/posterior pattern formation, camera-type eye development and eye-photoreceptors. In agreement with this analysis, the eye ‘master regulator’ *Pax6* emerges as the most central gene in cluster NC1 (Figs 5c and 6a), a notion that is further supported by its large number of predicted interactions (high out-degree).

The second cluster (Fig. 6a) resembles module M2 (Fig. 5c) comprising many OEP specific factors, which respond to FGF rapidly (e.g. 6 hrs: *Etv5*, *Foxi3*, *Gbx2* and *Hesx1)*. This cluster is therefore likely to represent the earliest phase of OEP induction. This is supported by the GO term analysis, which associates terms related to ear development and morphogenesis to this cluster (Fig. 6a).

Finally, the third cluster NC3 contains a mixture of genes (Fig. 6a), which characterise different tissues in the normal embryo, and overlaps with module M3 (Fig. 5c). It largely harbours late FGF response genes (12 or 24 hrs) including otic placode factors that are enhanced by FGF signalling (e.g. *Bmp4*, *Hey2*, *Sall1*, *Sall4*, *Six4*). In addition, NC3 also contains genes that are repressed by FGF (*Lef1*, *Geminin*, *Sox9*, *Gata3*, *Sox10*, *Irx3*, *Zfhx1b* and *Zic1*; Fig. 2b,d). Among these, *Geminin*, *Zic1* and *Zfhx1b* are expressed in the neural plate, but absent from the otic territory^50–52^, while *Sox9* and *Sox10* are present in neural crest cells^53, 54^ and only later in the otic placode (*Sox10*). The canonical Wnt target Lef1 also is among the FGF-repressed genes. It not only emerges as a central node based on betweenness centrality, but with a high out-degree is predicted to regulate many targets. Indeed, the Wnt pathway regulates the transition from OEP to committed otic cells^20, 55^ and our model suggests that Lef1 is a key player during this process. In summary, NC3 may represent a module that promotes otic character, while at the same time repressing alternative fates.

The fourth and the fifth cluster (Supplementary Fig. S3) correspond to genes at the periphery of the GENIE3 network with very few interactions with the central nodes (Fig. 5c). Overall, this analysis indicates that using time-series data to model a GRN generates distinct modules that appear to recapitulate normal development during otic induction. In addition, this approach also reveals new factors hitherto not linked to ear formation that may play a central role during otic commitment.

### Network predictions suggest new regulatory circuits to stabilise otic fate

The time course analysis described above reveals the genetic hierarchy downstream of FGF signalling as pPPR cells become specified as OEPs including both activated and repressed genes, while the GRN provides a global view of the network architecture that models this process. Our results reveal Etv5 and Pax6 as the only direct targets among the genes tested (Fig. 3), as well as a small cohort of transcripts whose expression is promoted or inhibited rapidly 6 hrs after FGF exposure (Fig. 2). Several of these have already been implicated in OEP specification (e.g. Foxi3, Gbx2, Six and Eya family members)^8, 44^ or in the acquisition of anterior placode fates (e.g. Pax6, SSTR5)^30^. However, how information is propagated through the network downstream of these factors to stabilise OEP identity and repress alternative fates is currently poorly understood. Here we use the predicted GRN to propose regulatory circuits by exploring the nearest neighbours of key FGF responsive genes and their predicted interactions.

#### Positive feedback loops stabilise the posterior PPR network downstream of FGF signalling

**Figure 7:**
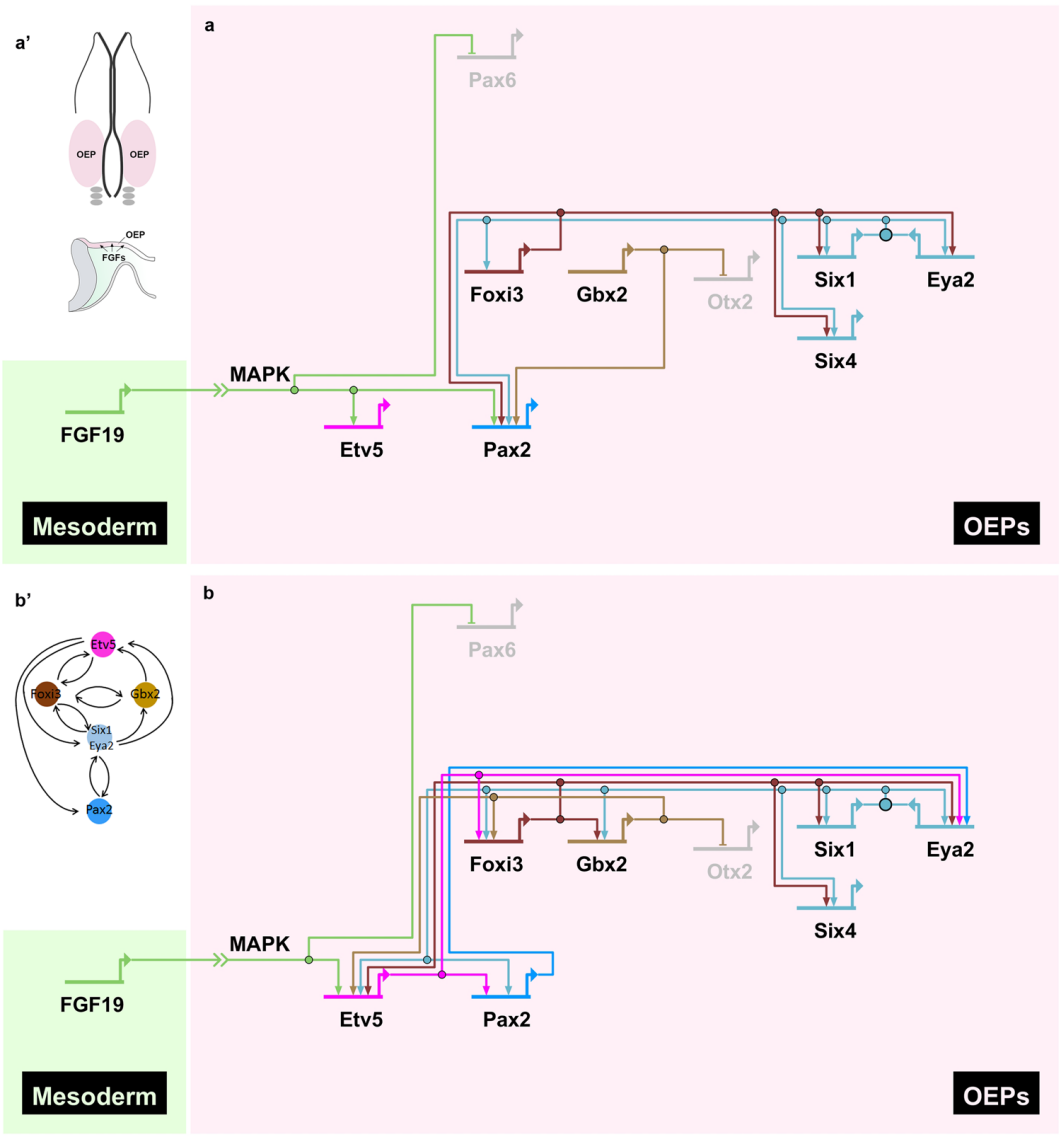
Network inference reveals a small FGF-activated circuit of positive feed-back loops. (**a**) Using published data (see Supplementary Table 1) a network of FGF-response genes during early OEP induction was generated using BioTapestry. Positive interactions are shown as arrows and repressive interactions as horizontal bars. Diagram of an embryo at OEP stage (a’, top) with the section (a’, bottom) showing the mesoderm as FGF source. (**b**) Network incorporating our data showing that Etv5 and Pax6 are direct FGF targets (see Fig. 3), as well as predicted interactions by GENIE3 network inference and first neighbour analysis (see Fig. S4). This reveals a small circuit of positive feed-back loops involving key OEP genes (b’). See text for details.

First, we briefly summarise the interactions of PPR and OEP transcription factors that have already been described. Members of the Six and Eya families are expressed in the entire PPR, while *Foxi3* and *Gbx2* are restricted to its posterior portion. Together, they provide crucial input for *Pax2*, one of the earliest genes labelling OEPs^8, 56–60^. Foxi1/3 and the Six1/Eya2 complex regulate each other in a positive feedback loop^8^, while Gbx2 is responsible to restrict *Otx2* anteriorly^44^. Downstream of these factors FGF initiates OEP specification: the FGF mediators *Etv4* and *Etv5* become expressed^41^ and *Pax2* expression is activated in response to FGF^9–11, 15, 18, 20, 32–35, 37–40, 60^ (Fig. 7a,a’).

Next we use our experimental time course and predictions to enrich the network upstream of *Pax2* (Fig. 7b,b’). Of the genes tested, Etv5 emerges as the only direct FGF target in OEPs (Fig. 3). Our network therefore assumes that Etv5 regulates all FGF responsive genes as the simplest explanation. We also show that *Foxi3* and *Gbx2* are under the control of FGF (Figs 2 and 4) i.e. downstream of Etv5. Analysis of their nearest neighbours (Supplementary Fig. S4) predicts that all three factors promote the expression of the others, either directly (Etv5 ↔ Foxi3; Foxi3 ↔ Gbx2) or indirectly (Etv5 ↔ Gbx2 via Foxi3). Likewise, Etv5 and Eya2 are predicted to form a positive feedback loop (Supplementary Fig. S4) linking FGF input and the maintenance of the Six/Eya complex at PPR stages. Our finding that *Eya2* maintenance requires FGF input (Fig. 4) supports this prediction. Finally, *Pax2* is known to be regulated by Foxi3 and Gbx2^8, 44, 59, 62, 63^, however our network analysis predicts that it is also activated directly by Etv5 (Supplementary Fig. S4). We therefore propose that a small circuit of positive feedback loops stabilises posterior PPR identity in response to FGF signalling (Fig. 7b’). Together FGF and the transcription factors within this circuit activate the OEP specific expression of *Pax2* to initiate the otic programme.

### Inhibitory loops refine gene expression in the otic placode

**Figure 8:**
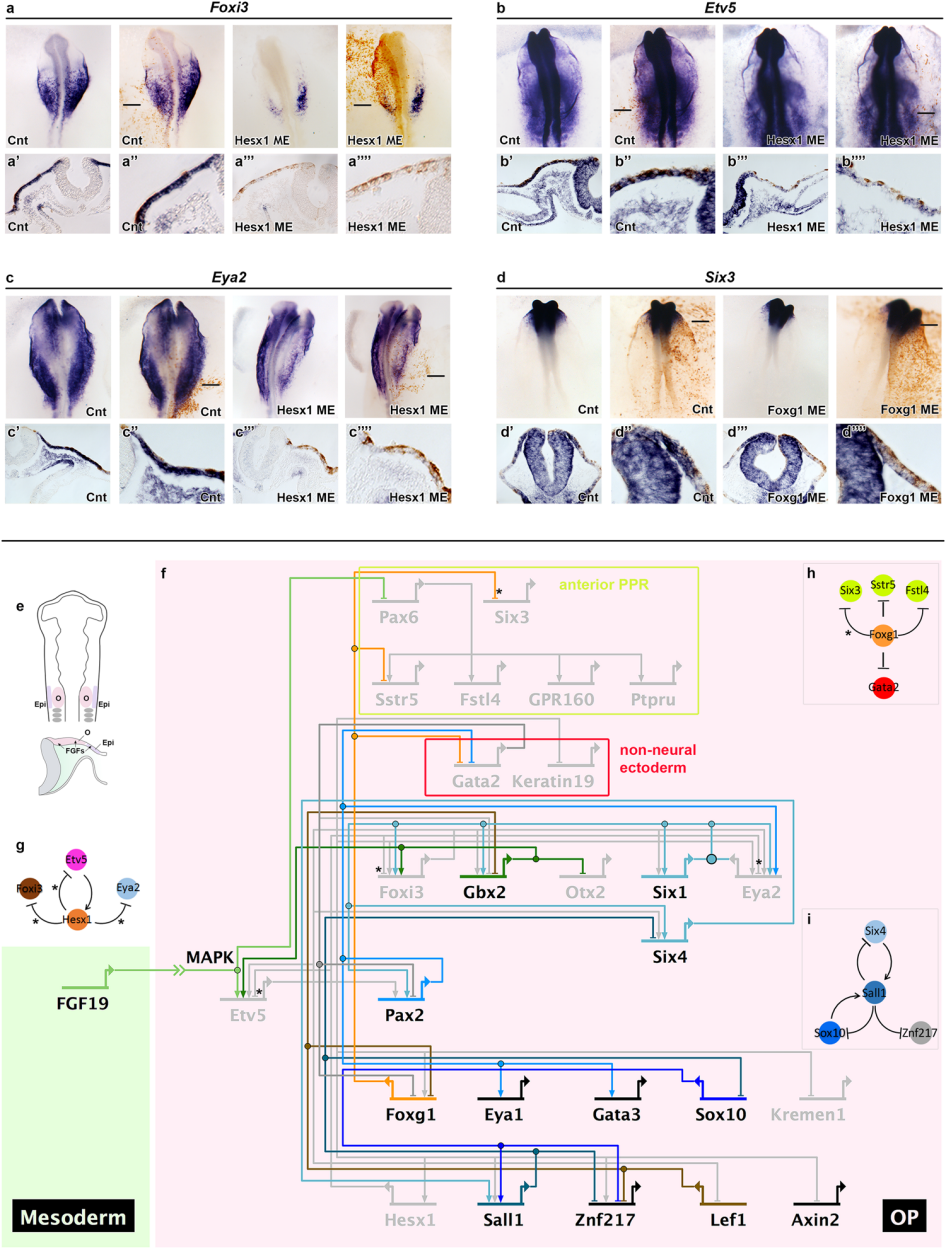
Network inference predicts inhibitory circuits to stabilise otic fate. (**a**) Misexpression (ME) of Hesx1 (2/5), but not of GFP (6/6) leads to loss of *Foxi3* expression in OEPs (a, a’- a””; blue) Note: normal *Foxi3* expression is very dynamic and changes rapidly; control embryo is at 10 ss and experimental embryo at 8 ss. (**b**,**c**) Hesx1 ME results in a reduction of *Etv5* (b, b’, b”; blue, 2/11) and *Eya2* (c, c’, c”; blue, 2/10) in OEPs, while controls do not show any loss (*Etv5*: 10/10; *Eya2:* 9/9). (**d**) Misexpression of Foxg1 (3/15), but not of GFP (11/11) in the anterior head region causes a reduction of *Six3* in the anterior PPR. For each marker, the two panels on the left are controls (Cnt) and those on the right represent Hesx1 or Foxg1 misexpression before (left) and after GFP immunostaining (right; brown) to visualise targeted cells. Panels below show sections through the same embryos; a’-d’, a’”-d’” low magnification; a”-d”, a””-d”” high magnification of the electroporated area. At 10 ss, otic (O) and epibranchial (Epi) fates have segregated (**e**) and new genes are activated downstream of FGF signalling among them the transcriptional repressors *Hesx1*, *Sall1*, *Znf217* and *Foxg1*. BioTapestry network incorporating the FGF time course data (Fig. 2), network predictions and first neighbour analysis (Fig. S4) and functional data (**f**). Hesx1 represses posterior PPR genes and the FGF mediator Etv5 (**g**) while Foxg1 inhibits the anterior PPR gene *Six3* and is predicted to repress other anterior and non-neural ectoderm transcripts (**h**). Sall1 is predicted to regulate *Sox10*, *Six4* and *Znf217* negatively (**i**). See text for details.

In the literature, few transcriptional interactions in the developing otic placode have been described. *Foxg1* is regulated by FGF signalling^23^, while Pax2 has been reported to control the expression of *Eya1* and *Gata3* ^64^. We show that prolonged FGF signalling (12 hrs) leads to the induction of a second set of genes (*Foxg1*, *Hesx1*, *Sall1*, *Znf217*), a time frame largely correlating well with their normal expression in the otic placode at 8–10 ss (Fig. 8). *Hesx1*, however, is normally only expressed at vesicle stages; thus FGF induces it prematurely in isolated explants. All four factors are transcriptional repressors^65–69^, which appear to initiate an inhibitory circuit that shuts down some early OEP genes and may limit the FGF response. Hesx1 is predicted to repress *Eya2* and *Foxi3* (Supplementary Fig. S4), which indeed are lost from the placode around the onset of *Hesx1* in the otic vesicle^43, 62, 70^. Our data suggest that Hesx1 also represses *Etv5*, and thus may modulate FGF activity later (Fig. 8b; Supplementary Fig. S4). To test these predictions, we misexpressed Hesx1 in the otic territory at HH6 prior to its normal onset (Supplementary Fig. S5) and assessed the expression of *Foxi3*, *Etv5* and *Eya2* at 9–11 ss. We find that all three transcripts are indeed reduced (Fig. 8a–c; *Foxi3*: 2/5, *Etv5*: 2/11, *Eya2*: 2/10 embryos express normal levels).

The transcriptional repressor Sall1 is predicted to inhibit the PPR gene *Six4* (Supplementary Fig. S4), whose otic expression declines over time, as well as the otic placode factor *Sox10*. *Sox10* expression depends on FGF input (Fig. 4c), although FGF is not sufficient for its induction, and also requires Etv4, Sox8 and cMyb, which bind to its otic enhancer^71^. Thus, Sall1 may act to prevent its premature expression in response to these factors. Interestingly, both Six4 and Sox10 are predicted to enhance *Sall1* expression thus forming a negative feedback loop (Supplementary Fig. S4, Fig. 8i).

Currently, nothing is known about the role of Znf217 in otic development. However, our predictions suggest that it is target of multiple repressive interactions from Sall1, Sox10 and the Wnt effector Lef1 (Supplementary Fig. S4, Fig. 8f). In summary, downstream of Pax2 and FGF, inhibitory loops appear to refine otic gene expression.

### Repressing alternative fates via Pax2 and Foxg1

In the PPR precursors for different placodes are initially intermingled, but also mixed with future epidermal and neural crest cells. The mechanisms that segregate cells of different fates are only beginning to emerge. Gbx2 and Otx2 mutually repress each other to separate otic and epibranchial progenitors from more anterior placodes^44^. Likewise, FGF signalling initiates the repression of lens specification^26^ and we confirm this finding with our FGF time course analysis (Figs 2 and 4). In addition, other anterior transcripts are rapidly repressed by FGF signalling (*Sstr5*, *Fstl4*, *GPR160*, *Ptpru*) and we suggest that Foxg1 may play a central role to maintain their repressed state: Foxg1 is predicted to inhibit the expression of *Sstr5* and *Fstl4*, as well as that of the lens/olfactory gene *Six3* (Supplementary Fig. S4, Fig. 8h). Indeed, misexpression of Foxg1 in the anterior PPR inhibits *Six3* expression (Fig. 8d; 3/15) confirming this prediction.

Likewise, several factors appear to cooperate to prevent epidermal gene expression. FGF inhibits epidermal *Keratin19* and *Gata2*, and this pathway is indeed required for their absence from otic cells (Figs 2 and 4). In addition, our network suggests that FGF acts through Foxg1 and Pax2 to repress *Gata2*, while conversely Gata2 is predicted to repress *Pax2*, *Foxg1*, *Gbx2* and *Etv5* (Supplementary Fig. S4, Fig. 8f). Thus, preventing Gata2 expression appears crucial to allow otic placode formation.

#### FGF signalling prevents premature activation of canonical Wnt signalling

While FGF activity is required for OEP induction, it must be switched off for cells to mature and acquire otic identity. Thereafter, canonical Wnt signalling promotes otic commitment^20, 22, 55^. Our data suggest that FGF plays a role in preventing premature activation of the Wnt pathway. *Axin2*, a readout for canonical Wnt activity, is rapidly upregulated when FGF is inhibited, and actively repressed after prolonged FGF exposure (Figs 2 and 4) as are *Lef1* and the Wnt co-receptor *Kremen1*. Our network analysis proposes that Foxi3 mediates FGF action, since it is predicted to repress *Lef1*. Conversely, Lef1 itself is predicted to inhibit several FGF dependent otic genes including *Foxg1*, *Gbx2* and *Znf217*, which are normally expressed before Wnt signalling becomes active in the otic placode (Supplementary Fig. S4, Fig. 8f). These results highlight how different signals modulate each other’s activity to control otic development and point to the regulatory circuits mediating this process.

## Discussion

The vertebrate inner ear arises from a pool of sensory progenitor cells that are initially competent to contribute to all sense organs and sensory ganglia in the head. Over time their potential is restricted, and cells next to the hindbrain become committed to the ear lineage. Rather than involving a single molecular switch ear commitment is achieved gradually as cells are exposed to different sequential signals^1, 28, 29^. FGF signalling is widely accepted as otic inducing signal: in the absence of FGF activity the otic placode does not form^10, 11, 15, 17, 18, 23, 32, 34, 40, 42, 72–78^ and when exposed to FGFs placode progenitors activate the otic-epibranchial programme^15, 32, 37–40, 77, 79–81^. Here we dissect the temporal hierarchy downstream of FGF signalling using a combination of experimental and network inference approaches. Our findings suggest that the main role of FGF during otic-epibranchial progenitor induction is to activate a small transcriptional circuit, which in turn may be sufficient to implement the ear programme autonomously.

Analysing the response of sensory progenitors to FGF over time reveals that rather than inducing many genes, FGF rapidly promotes the expression of a few transcription factors (*Etv5*, *Foxi3* and *Gbx2*), while others are initiated much later. *Etv5* appears to be the only direct FGF target of the genes tested placing *Foxi3* and *Gbx2* downstream of Etv5. All three factors are already expressed in sensory progenitors: FGF enhances, but does not induce their expression. It is possible that posterior PPR cells retain residual FGF activity and this may explain the transient upregulation of otic genes *in vitro*, in the absence of exogenous FGFs (Fig. 1). Network predictions indicate that *Etv5*, *Foxi3* and *Gbx2* perpetuate their own expression thus locking cells in a posterior PPR transcriptional state (Fig. 7b’). Foxi3 also forms a positive feedback loop with the PPR specifiers Six1 and Eya2^8, 62, 82^, and together they regulate the onset of *Pax2*, the earliest known OEP marker^8, 57, 58, 62, 63, 77, 80, 83, 84^. Together, our data suggest that once this circuit of positive feedback loops is established, cells are able to maintain their identity even in the absence of FGF signalling (Fig. 7b’). Indeed, OEPs are specified as soon as *Pax2* becomes expressed (4–5 ss): when cultured in isolation OEP explants continue to express otic specific genes and generate neurons in the absence of additional signals^24, 25^. We therefore suggest that the major role of FGF signalling during otic placode initiation is to activate a small sub-circuit of genes, whose role is to stabilise the OEP programme before additional signals commit cells to inner ear or epibranchial identity.

FGFs are critical to activate the OEP programme. However, it has been suggested that attenuation of the pathway is required for cells to become committed to the ear lineage, while continued signalling is necessary for epibranchial placodes to form^12, 16, 20^. Thus, FGF signalling may be tightly controlled and this may occur on multiple levels. *Sprouty1* and -*2* inhibit MAPK signalling downstream of the FGF receptor^85, 86^ and both become rapidly upregulated as the placode forms^87–89^. In their absence, the otic placode is enlarged and cells that normally contribute to the epidermis are now recruited into the placode suggesting that FGF inhibition is required to control otic placode size^87^. Our network analysis points to a second mechanism to regulate FGF signalling acting at vesicle stages. Downstream of the early OEP circuit, several transcriptional repressors are activated including *Hesx1* ^65, 66, 69^. We show that when expressed prematurely, Hesx1 represses the transcription factor *Etv5*, a direct target of FGF signalling (Fig. 8b) suggesting a possible role in limiting FGF signalling in the otic vesicle.

Having received FGF signalling OEPs are rapidly specified^25^ becoming independent of additional signals suggesting that transcriptional programmes must be in place to segregate otic cells from other ectodermal fates and to reinforce ear identity. Our network analysis and functional experiments demonstrate that Hesx1 not only represses *Etv5*, but also the posterior PPR genes *Foxi3* and *Eya2*. Indeed, both are downregulated as the otic placode matures and it is possible that this is required to maintain otic character. Likewise, the transcriptional repressor *Foxg1* is activated downstream of the OEP network. Network inference predicts Foxg1 to be key for repression of other fates (Fig. 8h), in particular anterior PPR derivatives like lens and olfactory placodes. We have previously shown that lens is the default state of all sensory progenitors and that FGF signalling initiates, but does not complete lens repression in non-lens ectoderm^26^. Here we show that the lens transcription factor *Pax6* is a direct target of FGF signalling and propose that Foxg1 continues to prevent its activation in the ear. Our network analysis predicts that *Foxg1* represses two different *Pax6* regulators: the somatostatin receptor *SSTR5* ^30^ and the transcription factor *Six3* which binds to the Pax6-lens enhancer^90^, and our functional data confirm *Six3* repression by Foxg1. Together, our data suggest that repressive loops are critical to ensure the progression of OEPs towards otic commitment, while simultaneously preventing alternative fates.

In summary, using a combination of time course analysis and network inference we describe a framework for understanding the regulatory logic that initiates ear development from sensory progenitors. Our gene network highlights the hierarchical organisation of otic induction and provides mechanistic insight into how signalling information is propagated through the network. We suggest that downstream of FGF signalling a few transcription factors form a circuit of positive feedback loops that is sufficient to maintain OEP identity and thus keeps cells competent to respond to the next signalling input.

## Methods

All experiments were carried out in accordance with the institutional guidelines and regulations.

### Embryo manipulation and pPPR explant culture

Experiments on chick embryos prior to E10 do not require a home office license or institutional approval and were carried out according to the institutional guidelines. Fertilized hens’ eggs were obtained from Winter Farm (Herts, UK) and incubated in a humidified incubator at 38 °C hours until reaching primitive streak or head fold stages. For culture, embryos were isolated using filter papers^91^, electroporated using 5 pulses of 4.7 V for 50 ms each with a 750 ms gap as previously described^92^ and maintained in filter paper culture until they had reached 9–12 ss. Foxg1overexpression was carried out at primitive streak stages and Hesx1 overexpression at head fold stages. For explant cultures, head fold stage embryos were isolated in Tyrode’s saline; the pPPR ectoderm with or without the underlying mesoderm were dissected and then cultured in collagen drops as described^26^. Culture medium and collagen were supplemented with FGF2 (0.25ng/μl; R&D), DMSO, SU5402 (10 μM; Tocris) or CHX (10 μM; Sigma). Tissues were cultured for 3, 6, 12 and 24 hours.

### NanoString nCounter

A NanoString probe set (Supplementary File 1) was designed containing known otic and other placode markers, known PPR, neural, neural crest and non-neural ectoderm markers and new placode genes identified in a recent microarray screen for new regulators in placode formation (unpublished). For each experimental condition, eight to ten explants were lysed in 5 μl of lysis buffer (Ambion). For each condition three independent experiments were carried out and analyzed by nCounter® Analysis System (Life Sciences) using a customized probe set of 126 genes. Total RNA was hybridized with capture and reporter probes at 65 °C over night. According to the nCounter Gene Expression Assay Manual the target/probe complexes were washed, immobilized, and data were collected by the nCounter Digital Analyzer. Data were analyzed following company instructions. A cut off of fold change >= 1.25 and <= 0.75 was used to identify upregulated and downregulated genes, respectively, in combination with a p-value <=0.05 (unpaired t-test). Full list of probes and their targeted sequences are in Supplementary File 1.

### Plasmids, antibodies and *in situ* hybridization

The following chick ESTs were used to generate Digoxigenin–labeled antisense probes: Chd7 ChEST757h23, Cxcl14 ChEST896P24, Fstl4 ChEST433o1, GPR160 ChEST21c16, Hey2 ChEST923p18, Homer2 ChEST795g2, Kremen1-like ChEST751a10, Mynn ChEST536f8, PTPRU ChEST714k5 and Tox3 ChEST1009p6. Etv5 was obtained from M. Bronner, Foxi3 from A. Groves, Gata2 and Lef1 from C.D. Stern, Pax2 from M. Golding and Pax6 from A. Bang. RNA probes were synthesized with T7, T3 or SP6 RNA polymerase (Roche). Whole mount or explant *in situ* hybridization was performed as described previously^93^. Mouse Foxg1 (a gift from C. Houart) and Hesx1 (a gift form J.P. Barbera-Martinez) were co-electroporated with eGFP. Electroporated embryos were processed for *in situ* hybridization followed by antibody staining for GFP (Invitrogen) using an HRP-coupled secondary antibody (Jackson) as previously described^92, 93^.

### Gene regulatory network (GRN) inference

GRN inference of the normalised NanoString data was carried out using GENIE3 R implementation. GENIE3 outperforms other popular inference methods on real and simulated data^94^ and shows excellent performance in previous studies^95, 96^. Before network inference, genes with very low expression values (<0.00004), which cannot be detected by *in situ* hybridization, were treated as absent and their values set to 0. As GENIE3 input, the normalised NanoString gene expression data and a list of transcription factors (potential regulators in the NanoString dataset) were used. GENIE3 produces a directed network by attempting to explain the expression profile of a target gene by the expression profiles of all input genes using a tree-based ensemble method: Random Forests. Then the importance of each input gene (regulator) in explaining the expression profile of a target gene is calculated by inferring a network *n* number of times (*n* = 1000 used in present study). The importance measure (IM) is then taken as an indication of a putative regulatory link. Following network inference, all regulatory links were ranked according to their IM with larger values indicating greater significance. To test the efficacy of the network, the predicted interactions were compared to 76 known interactions from the literature between PPR to otic placode stages using union and intersection functions in Cytoscape. After assessing the percentage of true positives retrieved (sensitivity) against the total number of predicted interactions, a threshold of 0.006 on the IM was considered optimal.

### Clustering

To identify groups of co-expressed genes in the NanoString data, normalised expression values of genes were used to perform hierarchical clustering (using Euclidean distance) and to generate heatmaps using the R package gplots^97^. To identify modules in the network, Community clustering^49^ was performed using the GLay plugin^98^ in Cytoscape. The advantage of Girvan and Newman’s clustering algorithm is that it does not require the number of clusters to be fixed as in other clustering techniques such as K-means. Thus, it allows finding the natural community structure within the network. Following clustering, the resulting modules were annotated with Gene Ontology (GO) and KEGG pathways using Cytoscape plugin BiNGO^99, 100^.

### Correlation analysis

To determine negative and positive relationships in GENIE3 predicted network, Pearson’s correlation coefficient was calculated between all pairs of genes in the NanoString data and displayed as a heatmap using the R package gplots^97^.

### Network display

The network was viewed and analysed in Cytoscape v 3.0.2^101^. Size and colour of the nodes were assigned according to the betweenness centrality (number of shortest paths between nodes in the network that pass through a particular node) or out-degree of each node in the network and the edges were weighted according to the IM values. The gene regulatory network models were drawn using BioTapestry^102–104^.

## Electronic supplementary material

Supplementary Information

Supplementarty data set 1

Supplementary data set 2

Supplementry data set 3
