# Supplementary material for: A gene network regulated by FGF signalling during ear development
Source: Sci Rep. 2017 Jul 21;7:6162. doi: 10.1038/s41598-017-05472-0 (PMC5522468; doi:10.1038/s41598-017-05472-0)
Supplement: Supplementary file 1 — Supplementary Information [file 41598_2017_5472_MOESM1_ESM.doc]

**A gene network regulated by FGF signalling during ear development**

Maryam Anwar, Monica Tambalo, Ramya Ranganathan, Timothy Grocott and Andrea Streit

**Supplementary Figure 1**

**
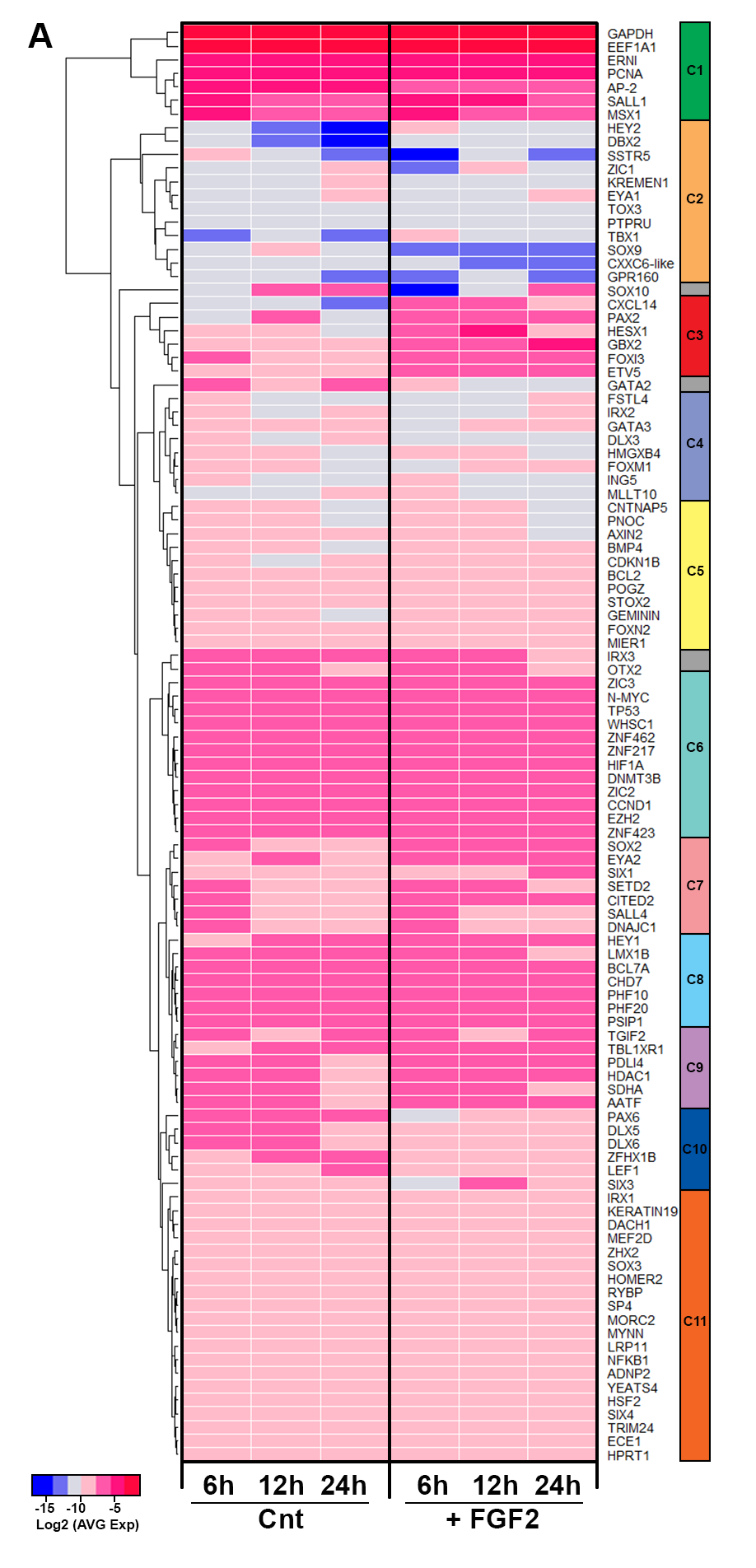
**

**Supplementary Figure S1. Hierarchical clustering of NanoString data reveals co-expressed genes**

Posterior PPR explants were cultured in the absence or presence of FGF2 and gene expression was assessed by NanoString. (**A**) Hierarchical clustering of the Log2 (average expression level) of genes in Control (Cnt) and +FGF2 at 6,12 and 24 hrs reveals 11 clusters. Low levels of expression are shown in blue, whereas high levels of expression are shown in a gradient of pink.

**Supplementary Figure 2**

**
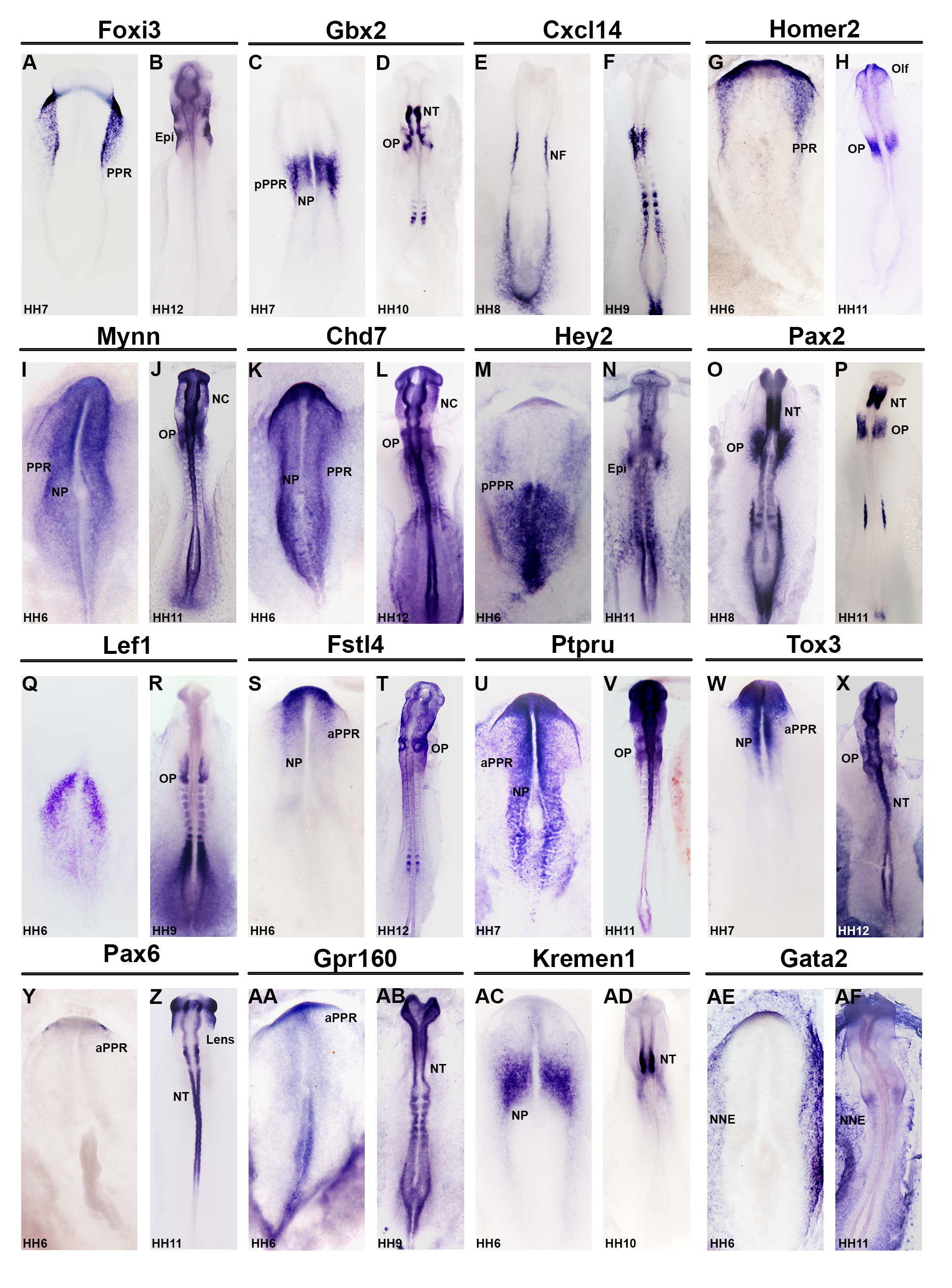
**

**Supplementary Figure S2. Expression pattern of FGF responsive genes**

In general, FGF induced genes are expressed in the PPR and/or in the otic placode. *Foxi3*, is enriched in the PPR at neural plate stage (HH6) (**A**) and later becomes restricted to the epibranchial placodes (**B**). *Gbx2* is expressed in the posterior PPR (**C**) and later in the ridge of the otic cup (**D**). The chemokine *Cxcl14* is found at the medial edge of the developing otic territory (**E**, **F**). The scaffold protein *Homer2* is expressed in the entire PPR (**G**) and is then enriched in the olfactory and otic placodes (**H**). The transcription factor *Mynn* and the chromatin-remodelling enzyme *Chd7* are initially widely expressed in the ectoderm (**I**, **K**) to become enriched in the otic placode, neural crest and neural tube (**J**, **L**). *Hey2* is found in the posterior PPR (**M**) and in the nodose placode (**N**). The transcription factor *Pax2* is present in the otic-epibranchial territory (**O**, **P**). A different set of genes is not expressed in the posterior PPR and is initially repressed by FGF signalling e.g. *Lef1*, *Fstl4*, *Ptpru*, *Tox3* (**Q**, **S**, **U**, **W**). Later, all are expressed in the otic territory, and are positively regulated by FGF (**R**, **T**, **V**, **X**). The last class of genes includes FGF repressed genes. *Pax6* is expressed in the lens territory (**Y**, **Z**). *Gpr160* and *Kremen1* are mainly found in the neural plate (**AA**, **AB**, **AC**, **AD**), while *Gata2* is expressed in the non-neural ectoderm (**AE**, **AF**). PPR: pre-placodal region; aPPR: anterior pre-placodal region; pPPR: posterior pre-placodal region; NC: neural crest; NF: neural folds; NP: neural plate; OP: otic placode; Epi: epibranchial placode; Olf: olfactory placode; Lens: lens placode; NT: neural tube.

**Supplementary Figure 3**

**
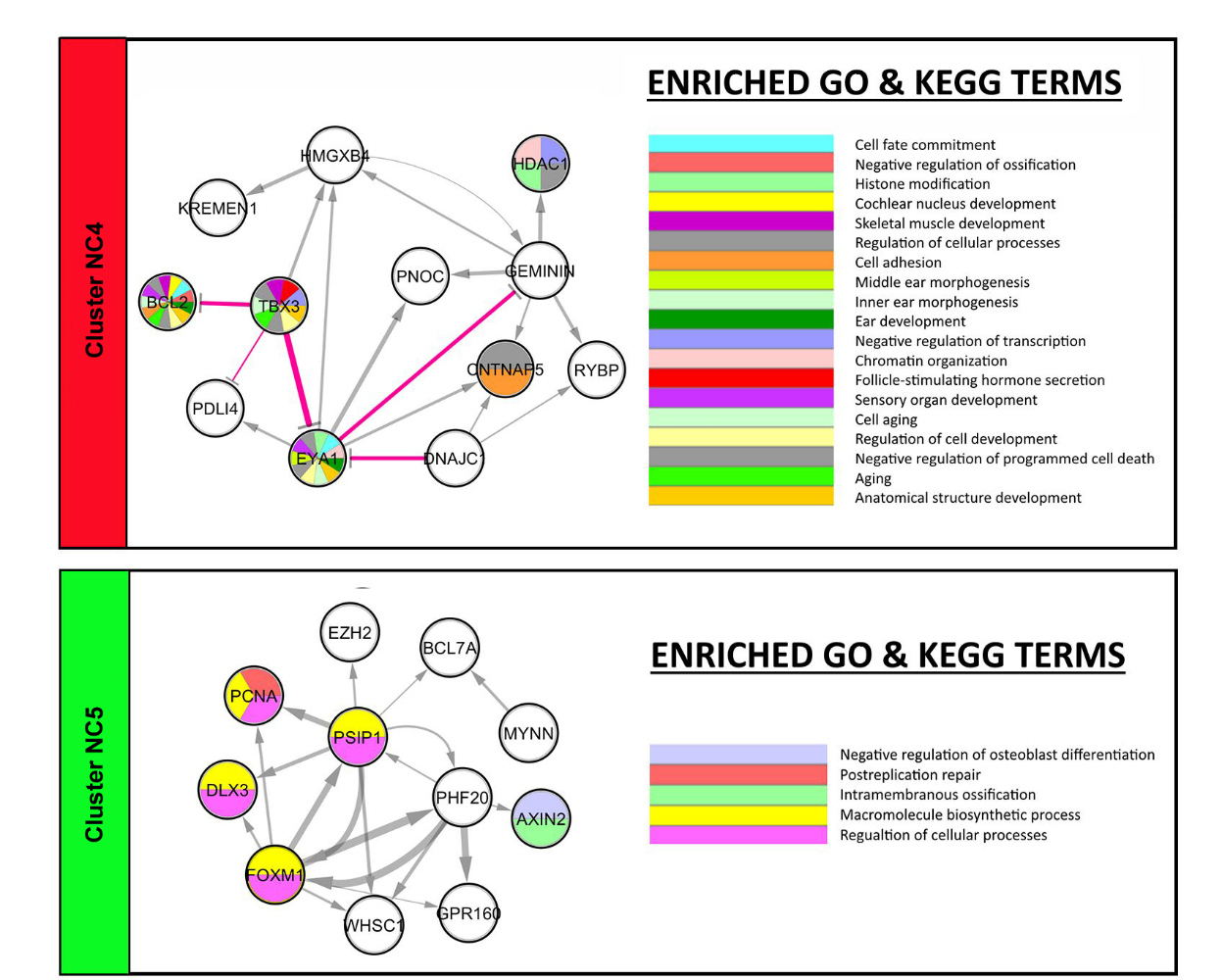
**

**Supplementary Figure S3. Newman’s community clusters**

Interactions and GO and KEGG term analysis (P-value <0.05) of clusters NC4 and NC5 generated by using Newman’s community clustering of the of the top 500 interactions in the predicted NanoString network.

**Supplementary Figure 4**

**
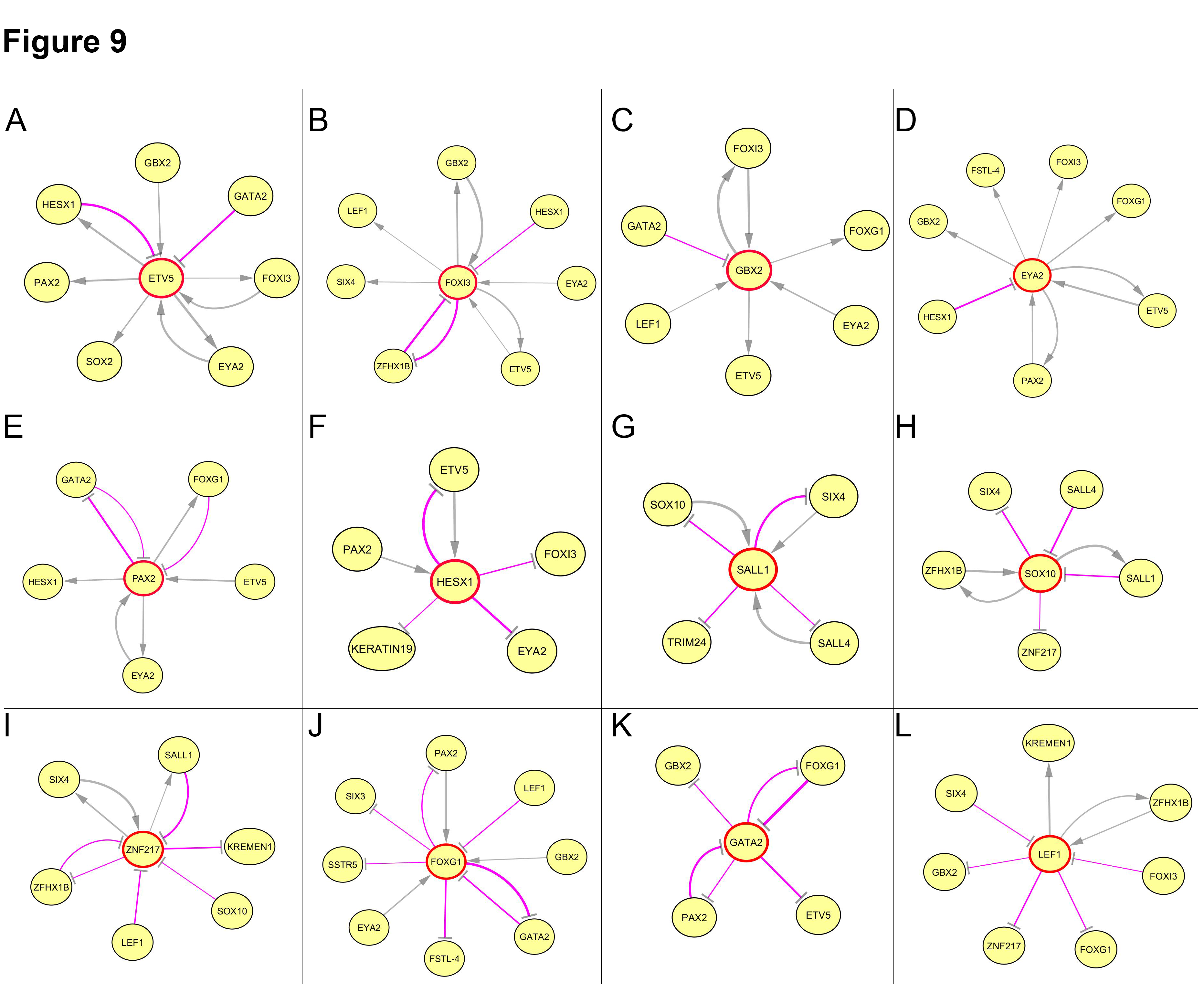
**

**Supplementary Figure S4. First neighbour analysis of key PPR, OEP and otic genes**

The first neighbours of PPR, OEP and otic genes (encircled in red) were extracted from the top 500 GENIE3 predictions and plotted in Cytoscape (**A-L**). Repressive (pink) and activating interactions were identified using Pearson’s correlation coefficient (see Figure 6B).

**Supplementary Figure 5**

**
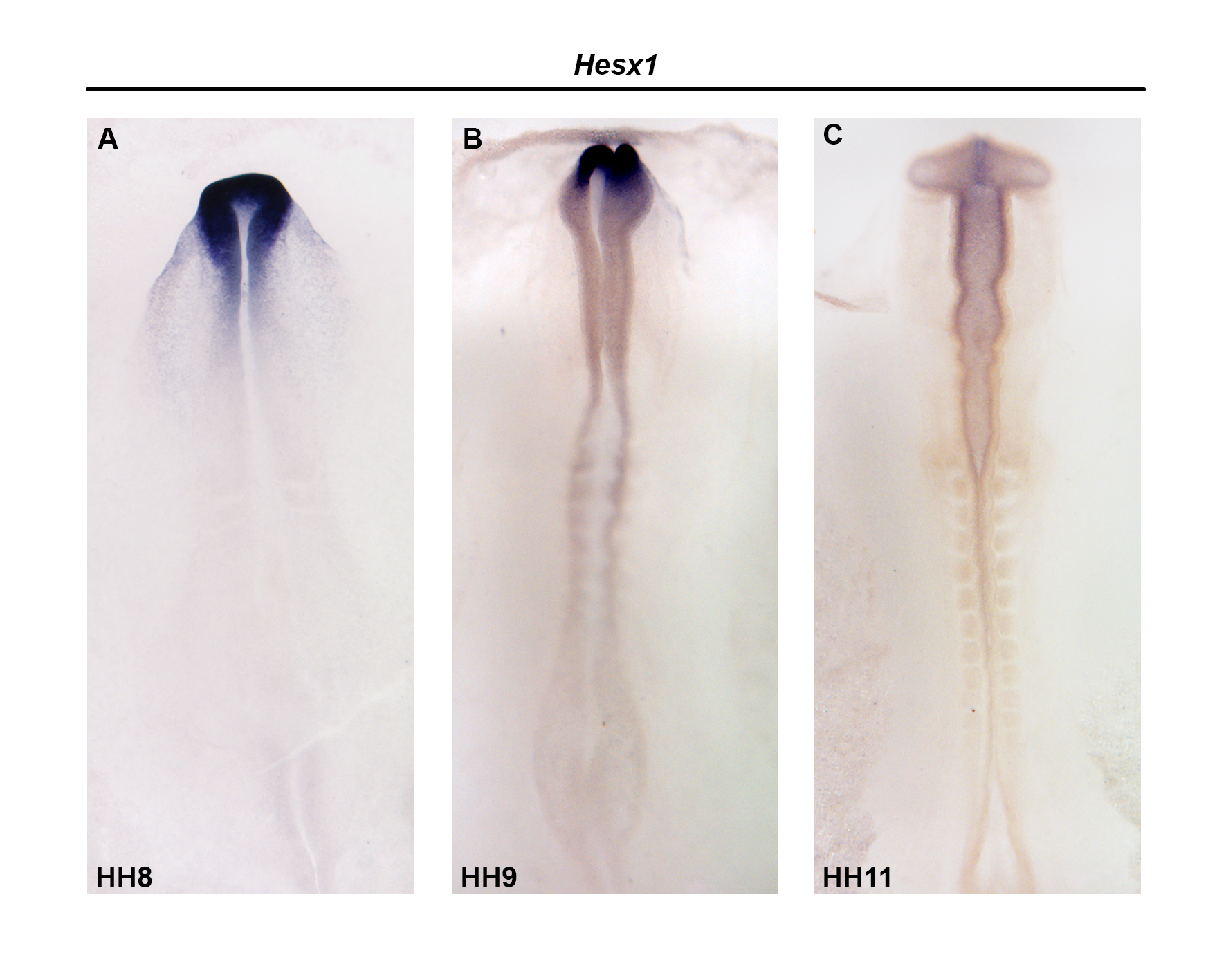
**

**Supplementary Figure S5. Expression pattern of *Hesx1*.**

*Hesx1* is expressed in the anterior neural fold and forebrain, but absent from the prospective otic vesicle (**A-C**). It begins to be expressed around Hamburger and Hamilton stage 17 at vesicle stages (not shown; see also geisha.arizona.edu).

**Supplementary Table 1. Genetic interactions at OEP stages.**

Summary of interactions from the literature.

| **Source** | **Interaction** | **Target** | **System** | **Evidence** |
| --- | --- | --- | --- | --- |
| **FGFs** | **Through** | **MAPK** | Chick | 1 |
| **FGFs** | **Promotes** | **Etv5**  **(Erm)** | Chick,  *Xenopus,*  Zebrafish | 2-4 |
| **FGFs (via Etv5), Six/Eya*** | **Promotes** | **Foxi3** | Chick,  *Xenopus,* Zebrafish | 5,6 |
| **Gbx2** | **Represses** | **Otx2** | *Xenopus* | 7 |
| **Foxi3, Six/Eya** | **Promotes** | **Six1** | Chick | 6,8 |
| **FGFs (via Etv5)*, Foxi3, Six/Eya** | **Promotes** | **Eya2** | Chick | 6,8,9 |
| **Foxi3*, Six/Eya** | **Promotes** | **Six4** | Chick,  *Xenopus* | 8,10 |
| **FGFs** | **Represses** | **Pax6** | Chick | 11 |
| **Gbx2, Foxi3, FGFs (via Etv5)*, Six1/Eya*** | **Promote** | **Pax2** | Chick, mouse, *Xenopus,*  zebrafish | 5-8,12-24 |
| **FGFs (via Etv5), Foxi3, Pax2** | **Promote** | **Foxg1** | Chick,  mouse | 1,6,25,26 |
| **Pax2** | **Promotes** | **Eya1** | Chick | 27 |
| **Pax2** | **Promotes** | **Gata3** | Chick | 27 |

* Interactions shown experimentally and from the top 500 predictions (GENIE3)

**References for Supplementary Table 1**

1 Yang, L. *et al.* Analysis of FGF-dependent and FGF-independent pathways in otic placode induction. *PloS one* **8**, e55011 (2013).

2 Roehl, H. & Nusslein-Volhard, C. Zebrafish pea3 and erm are general targets of FGF8 signaling. *Current biology : CB* **11**, 503-507 (2001).

3 Raible, F. & Brand, M. Tight transcriptional control of the ETS domain factors Erm and Pea3 by Fgf signaling during early zebrafish development. *Mechanisms of development* **107**, 105-117 (2001).

4 Lunn, J. S., Fishwick, K. J., Halley, P. A. & Storey, K. G. A spatial and temporal map of FGF/Erk1/2 activity and response repertoires in the early chick embryo. *Developmental biology* **302**, 536-552 (2007).

5 Phillips, B. T., Storch, E. M., Lekven, A. C. & Riley, B. B. A direct role for Fgf but not Wnt in otic placode induction. *Development* **131**, 923-931 (2004).

6 Khatri, S. B., Edlund, R. K. & Groves, A. K. Foxi3 is necessary for the induction of the chick otic placode in response to FGF signaling. *Developmental biology* **391**, 158-169 (2014).

7 Steventon, B., Mayor, R. & Streit, A. Mutual repression between Gbx2 and Otx2 in sensory placodes reveals a general mechanism for ectodermal patterning. *Developmental biology* **367**, 55-65 (2012).

8 Christophorou, N. A., Bailey, A. P., Hanson, S. & Streit, A. Activation of Six1 target genes is required for sensory placode formation. *Developmental biology* **336**, 327-336 (2009).

9 Litsiou, A., Hanson, S. & Streit, A. A balance of FGF, BMP and WNT signalling positions the future placode territory in the head. *Development* **132**, 4051-4062 (2005).

10 Kwon, H. J., Bhat, N., Sweet, E. M., Cornell, R. A. & Riley, B. B. Identification of early requirements for preplacodal ectoderm and sensory organ development. *PLoS genetics* **6**, e1001133 (2010).

11 Bailey, A. P., Bhattacharyya, S., Bronner-Fraser, M. & Streit, A. Lens specification is the ground state of all sensory placodes, from which FGF promotes olfactory identity. *Dev Cell* **11**, 505-517 (2006).

12 Leger, S. & Brand, M. Fgf8 and Fgf3 are required for zebrafish ear placode induction, maintenance and inner ear patterning. *Mechanisms of development* **119**, 91-108 (2002).

13 Maroon, H. *et al.* Fgf3 and Fgf8 are required together for formation of the otic placode and vesicle. *Development* **129**, 2099-2108 (2002).

14 Solomon, K. S. & Fritz, A. Concerted action of two dlx paralogs in sensory placode formation. *Development* **129**, 3127-3136 (2002).

15 Nissen, R. M., Yan, J., Amsterdam, A., Hopkins, N. & Burgess, S. M. Zebrafish foxi one modulates cellular responses to Fgf signaling required for the integrity of ear and jaw patterning. *Development* **130**, 2543-2554 (2003).

16 Solomon, K. S., Kudoh, T., Dawid, I. B. & Fritz, A. Zebrafish foxi1 mediates otic placode formation and jaw development. *Development* **130**, 929-940 (2003).

17 Wright, T. J. & Mansour, S. L. Fgf3 and Fgf10 are required for mouse otic placode induction. *Development* **130**, 3379-3390 (2003).

18 Hans, S., Liu, D. & Westerfield, M. Pax8 and Pax2a function synergistically in otic specification, downstream of the Foxi1 and Dlx3b transcription factors. *Development* **131**, 5091-5102 (2004).

19 Solomon, K. S., Kwak, S. J. & Fritz, A. Genetic interactions underlying otic placode induction and formation. *Developmental dynamics : an official publication of the American Association of Anatomists* **230**, 419-433 (2004).

20 Bricaud, O. & Collazo, A. The transcription factor six1 inhibits neuronal and promotes hair cell fate in the developing zebrafish (Danio rerio) inner ear. *The Journal of neuroscience : the official journal of the Society for Neuroscience* **26**, 10438-10451 (2006).

21 Padanad, M. S. & Riley, B. B. Pax2/8 proteins coordinate sequential induction of otic and epibranchial placodes through differential regulation of foxi1, sox3 and fgf24. *Developmental biology* **351**, 90-98 (2011).

22 Freter, S., Muta, Y., Mak, S. S., Rinkwitz, S. & Ladher, R. K. Progressive restriction of otic fate: the role of FGF and Wnt in resolving inner ear potential. *Development* **135**, 3415-3424 (2008).

23 Ladher, R. K., Anakwe, K. U., Gurney, A. L., Schoenwolf, G. C. & Francis-West, P. H. Identification of synergistic signals initiating inner ear development. *Science* **290**, 1965-1967 (2000).

24 Sun, S. K. *et al.* Epibranchial and otic placodes are induced by a common Fgf signal, but their subsequent development is independent. *Developmental biology* **303**, 675-686 (2007).

25 Freter, S. *et al.* Pax2 modulates proliferation during specification of the otic and epibranchial placodes. *Developmental dynamics : an official publication of the American Association of Anatomists* **241**, 1716-1728 (2012).

26 Urness, L. D., Paxton, C. N., Wang, X., Schoenwolf, G. C. & Mansour, S. L. FGF signaling regulates otic placode induction and refinement by controlling both ectodermal target genes and hindbrain Wnt8a. *Developmental biology* **340**, 595-604 (2010).

27 Christophorou, N. A., Mende, M., Lleras-Forero, L., Grocott, T. & Streit, A. Pax2 coordinates epithelial morphogenesis and cell fate in the inner ear. *Developmental biology* **345**, 180-190 (2010).

**Supplementary Dataset Files**

**Supplementary Dataset File 1.** Nanostring data files.

File containing the experimental data for all experiments analysed by NanoString.

**Supplementary Dataset File 2.** Top 500 interactions as predicted by GENIE3.

File containing the top 500 interactions as predicted from all NanoString data by GENIE3.

**Supplementary Dataset File 3.** Nanostring probe set.

File containing the gene names, gene IDs and targeted sequences for all the genes present in the Nanostring probe set.
